# Hierarchical Structure, Gelatinization, and Digestion Characteristics of Starch from Longan (*Dimocarpus longan* Lour.) Seeds

**DOI:** 10.3390/molecules23123262

**Published:** 2018-12-10

**Authors:** Ziman Hu, Lei Zhao, Zhuoyan Hu, Kai Wang

**Affiliations:** College of Food Science, South China Agricultural University, 483, Wushan Road, Guangzhou 510642, China; tannin_hu@163.com (Z.H.); scauzl@scau.edu.cn (L.Z.); zyhu@scau.edu.cn (Z.H)

**Keywords:** longan seed, starch, structure, gelatinization, in vitro digestion

## Abstract

Starch was isolated from longan seeds of three widely distributed cultivars (Chuliang, Shixia, and Caopu) in China. Comparisons of the multi-level structure of the starch of longan seeds among various cultivars were made, and the relations between these structural and property characteristics are discussed. The isolated starch, accounting for 44.9–49.5% (*w*/*w*) in longan seeds, had an oval or an irregular polygonal shape with a smooth surface. Their chain-length distributions (CLDs) varied with longan cultivar; Chuliang showed a larger proportion of longer amylopectin chains with a degree of polymerization (DP) 30~100. This is attributed to the slightly higher relative crystallinity of Chuliang longan seed starch. Apparent differences were also detected in amylose structure. Caopu showed a higher amylose content than Chuliang and Shixia, resulting in its lower gelatinization temperatures and enthalpy change. All longan seed starch had a typical A-type crystal structure with relative crystallinity ranging 28.6–28.9%. For raw starch, Caopu showed the lowest digestion rate, followed by Chuliang; Shixia showed the highest. This is because Caopu had the highest amylose content. Chuliang had a more intact structure than Shixia, as suggested by its higher crystallinity, although they had similar amylose content. After being fully gelatinized, all starch showed a similar digestion process, indicating that the digestibility of gelatinized starch does not differ with starch source or structure.

## 1. Introduction

Longan (*Dimocarpus longan* Lour.), belonging to the Sapindaceae family, is a prevalent fruit widely distributed in subtropical areas such as China, Thailand, Malaysia, and Vietnam [1]. *Dimocarpus longan* cv. Chuliang, Shixia, and Caopu are the predominant ones cultivated in China. Longan seeds are normally considered as agricultural waste in longan fruit processing, resulting in incredible resource waste and environmental pressure on a large scale. In recent years, more and more attention has been directed to longan seeds because they contain several bioactive compounds, including polysaccharides, phenolic compounds, and flavonoids, etc., and exhibit multiple functional properties, such as antioxidant, anti-inflammatory, anticancer, and anti-tyrosinase effects [2,3,4,5]. Starch in longan seeds account for about 60%, as reported by Xiao, et al. [6]; however, it has long been overlooked, lacking in-depth studies. Recently, Guo, et al. [7] studied the structure and functional properties of starch from five fruit kernels including longan. However, similarities and differences in longan seed starch from different cultivars have not yet been elucidated, and relations between the hierarchical structure and properties are not yet fully understood.

Starch is one of the most abundant biopolymers in nature and serves mainly as energy storage in plants. It is a major component of the human diet, supplying more than half of the energy for the human body. It is also an important industrial material that is widely used in paper, renewable packaging, textile, and pharmaceutical products, etc. Starch is composed of D-glucose units connected linearly by α-(1 → 4) linkages as backbones and by α-(1 → 6) linkages at branching points. As a complex macromolecule, starch has a multiple-level structure [8]. In addition, starch from different sources have distinct structural features, which leads to variations in their properties. Understanding the relationships between starch structure and properties is critical for the application of starch in food and non-food products [9]. Oates and Powell [10] studied the structural properties, including granule shape, amylose content, solubility, gelatinization, and enzyme hydrolysis properties of starch from tropical fruit seeds, such as longan, durian, jackfruit, mango, and rambutan, and found apparent differences in the starch from the various botanical sources. However, comparisons of the multi-level structure and properties of longan seed starch from different varieties, as well as the relationships between their structure and digestion characteristics, have not been studied yet.

In this study, seeds from three widely distributed longan cultivars (Chuliang, Shixia, and Caopu) in China were used as raw materials. Starch was isolated from the longan seeds and compared with normal maize starch. The multi-level structure of the starch, including granular, crystalline, and molecular structures, was characterized and these structural features were used to explain differences in their gelatinization and in vitro digestion properties. The results of this study will provide guidance on the application of longan seed starch in food and non-food products, and thus will be beneficial for the development of the global longan industry.

## 2. Results and Discussion

### 2.1. Chemical Composition of Longan Seeds

The content of starch, protein, and fat in longan seeds from three longan cultivars are summarized in Table 1. The results showed that longan seeds had about 44.9–49.5% (*w*/*w*) of starch, 6.0–8.1% (*w*/*w*) of protein, and 2.0–3.4% (*w*/*w*) of fat. The starch content among the longan seeds from the three cultivars was not significantly different, indicating that the genetic background and growth environment of longan does not have a significant influence on the accumulation of starch in longan seeds. In addition, significant differences were observed in the protein and fat content among the three cultivars: Shixia showed the highest protein content while Chuliang had the highest fat content.

### 2.2. Granular Structure

**Figure 1:**
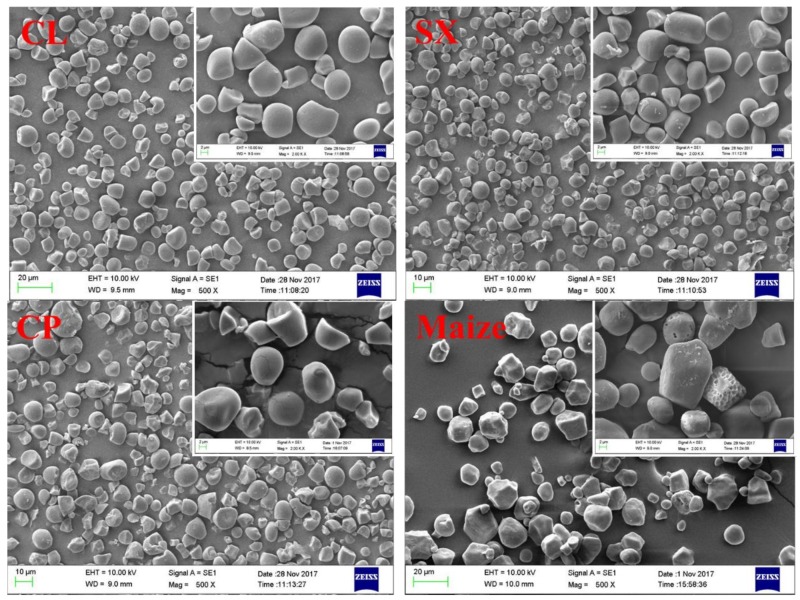
Scanning electron microscope images of starch from longan seeds, including Chuliang (CL), Shixia (SX), and Caopu (CP), compared with normal maize starch at 200× and 500× magnification.

The morphology of the starch granules isolated from the longan seeds, as compared with normal maize starch, was characterized using scanning electron microscopy (SEM), with images shown in Figure 1. In accordance with the literature, normal maize starch showed an oval and polygonal shape with sharp edges and pores on the surface, and most granules were below 20 μm in size [11,12]. Longan seed starch, in comparison, showed a similar granule shape to normal maize starch, with most granules being an oval or irregular polygonal shape. No pores were observed on the surface of the longan seed starch, which is different from normal maize starch. However, some of the longan seed starch granules had fissures on the surface, which might be a distinct characteristic of longan seed starch or could have resulted from the starch isolation process. The granular size of longan seed starch was slightly smaller than that of normal maize. All seed starch from the three longan cultivars were relatively similar in both granule shape and size, suggesting that genetic diversity and growth environmental conditions of various longan cultivars do not have a significant influence on the granular structure of the starch of longan seeds.

### 2.3. Crystalline Structure

**Figure 2:**
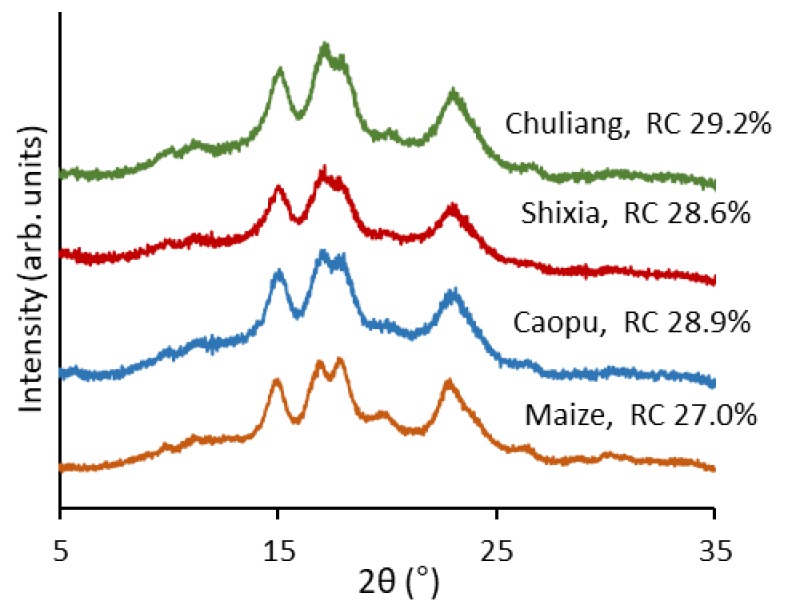
X-ray diffraction patterns and relative crystallinity (RC) of longan seed starch as compared with normal maize starch.

The crystalline structure of starch was analyzed using X-ray diffraction (XRD), and the diffraction patterns are displayed in Figure 2. All longan seed starch had an A-type crystalline structure with the main diffraction peaks at around 15, 17, 18, and 23° 2*θ*, which is the same as the crystalline type of normal maize starch [13]. The relative crystallinity of the starch from the seeds of the three longan cultivars was slightly higher than that of normal maize starch (27.0%), with Chuliang having the highest value (29.2%) followed by Caopu (28.9%) and Shixia (28.6%). 

### 2.4. Molecular Structure

**Figure 3:**
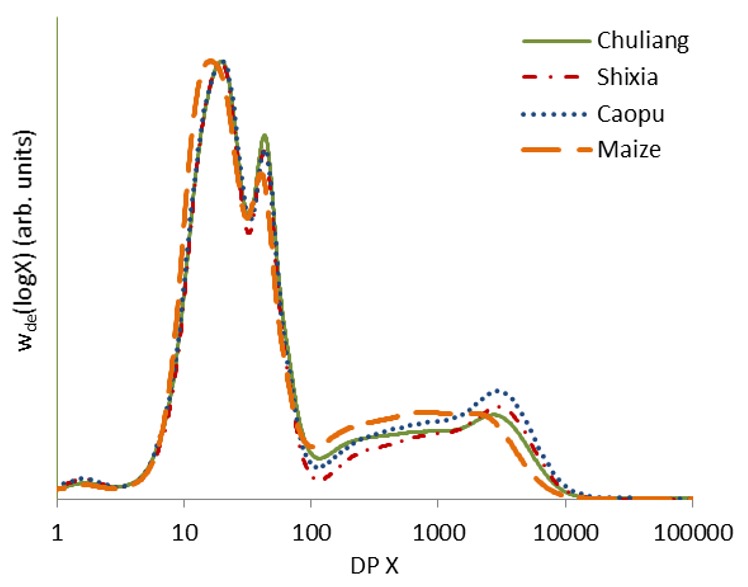
Chain-length distributions of longan seed starch compared with normal maize starch.

The chain-length distributions (CLDs) of all the starch samples were determined by size exclusion chromatography (SEC) after fully debranching the side chains of starch using isoamylase. The results are expressed as the SEC weight distribution of glucan chains from debranched starch as a function of DP (X), denoted as w_de_(logX) in Figure 3. To allow quantitative comparison among samples, all the CLDs were normalized to achieve the same height of the highest peak. Typical CLDs were observed for all the starch samples, with two amylopectin peaks at degree of polymerization (DP) X < 100 and multiple small bumps at DP > 100 [14]. As described previously, starch chains with DP < 100 are normally considered as amylopectin chains, while those with DP larger than 100 are predominantly amylose [15,16]. Therefore, the amylose content was calculated as the ratio of the area under amylose distribution of each sample to that of the whole distribution (Table 2).

As can be seen from Figure 3, the starch from all the three longan seed types showed two peaks at about DP 20 and 43 in the amylopectin region. The two peaks covered chains ranging DP < 30 and 30–100, respectively, and correspond to shorter amylopectin chains that stay within one crystalline/amorphous lamella (normally A and B_1_ chains) and longer amylopectin chains that span at least two lamellae (B_2_ and longer chains), respectively [14]. Small differences were observed in the amylopectin CLDs with Chuliang showing a slightly higher peak at DP 43, which indicates its higher proportion of longer amylopectin chains with DP 30–100. Normal maize starch, in comparison, was lower than the other starch at this peak, suggesting its lower proportion of longer amylopectin chains. The length of amylopectin chains has been found to be associated with the relative crystallinity of starch [13]. The higher relative crystallinity of Chuliang longan seed starch (shown in Figure 2) might be a result of its higher proportion of longer amylopectin chains with the degree of polymerization (DP) ranging from 30 to 100.

The amylose CLDs were different among the three longan cultivars. The highest amylose content was detected in Caopu (30.1%), followed by normal maize starch (29.1%, shown in Table 2). Although a similar content of amylose was observed in Chuliang (26.7%) and Shixia (26.6%), their amylose CLDs were different. Chuliang amylose consisted of a larger proportion of shorter chains with DP 100–1000 and a smaller proportion of longer chains with DP > 1000 than Shixia. Caopu amylose exhibited a higher proportion of longer amylose chains while normal maize starch was rich in short chains and lacked long chains in comparison. Differences in the molecular structure most likely result from the genetic background, as well as environmental factors of each longan cultivar, and these characteristics may also have an impact on the higher-level structure and properties of the starch.

### 2.5. Thermal Properties

Starch gelatinization corresponds to the dissociation of amylopectin double helices from a semi-crystalline structure to an amorphous conformation. The gelatinization temperatures, including onset (T_o_), peak (T_p_), and conclusion (T_c_) temperatures and enthalpy change (∆H) of seed starch from three longan cultivars were determined using a differential scanning calorimeter (DSC), and the results are summarized in Table 2. Starch gelatinization peaks were detected within a narrow range from 72.3 to 88.3 °C, and the enthalpy change ranged from 13.5 to 16.4 J/g. Among the three longan cultivars, significant differences were observed in the gelatinization temperatures (T_o_, T_p_, and T_c_) of the seed starch, with Chuliang showing the highest gelatinization temperatures and Caopu exhibiting the lowest. The enthalpy changes of longan seed starch showed a similar trend. Chuliang and Shixia displayed higher enthalpy change values (16.4 and 15.6 J/g, respectively) than Caopu (13.5 J/g).

Starch gelatinization properties could be influenced by multiple factors including the granule morphology, crystalline structure, amylose content, and molecular structure of the starch, etc. [9]. In the current study, seed starch from three longan cultivars were similar in their granule morphology and crystalline type. However, apparent differences were observed in their amylopectin CLD, crystallinity, and amylose content. From data shown in Table 2 and Figure 3, the gelatinization properties (including T_o_, T_p_, T_c_, and ∆H) were not correlated with the amylopectin CLD nor the crystallinity of the starch. Therefore, differences in the gelatinization temperatures of the three longan seed starch samples most likely resulted from variations in the amylose content. This is in accordance with previously published results showing that starch gelatinization temperatures and enthalpy change are negatively related to amylose content [17,18]. In addition, these results also confirmed that the enthalpy change has no direct correlation with the crystallinity of starch because the enthalpy change results mainly from the disruption of the double helices inside the starch molecule instead of the destruction of the crystalline order [19].

### 2.6. In Vitro Starch Digestibility

**Figure 4:**
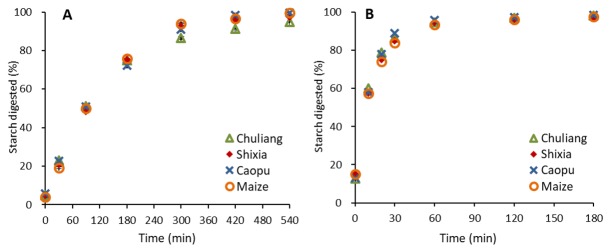
Digestogram of raw (**A**) and freshly gelatinized (**B**) longan seed starch as compared with normal maize starch.

The isolated longan seed starch samples were enzymatically digested by pancreatin and amyloglucosidase in vitro to stimulate their digestion process in food. Although starch is normally consumed after cooking, it sometimes cannot be fully gelatinized because of factors such as food processing conditions or lack of water in food systems, etc. Therefore, the in vitro starch digestion properties of longan seed starch, as compared with normal maize starch, were evaluated in both raw and freshly gelatinized states in this study. The resulting digestion curves are exhibited in Figure 4. All digestion curves were well-fitted with first-order kinetics, and the obtained digestion rate coefficients k are listed in Table 3.

As shown in Figure 4A, all raw starch samples showed similar digestion trends with a rapid digestion phase in the first 2 h of digestion followed by a slower digestion phase until a plateau was achieved at about 8 h of digestion. Slight differences were observed in the digestion curves of the raw longan seed starch. The digestion rate coefficient values (summarized in Table 3) showed the digestion rate of Shixia seed starch was the highest, and it was similar to that of normal maize starch. Chuliang seed starch showed a significantly lower digestion rate, while Caopu showed the lowest.

The digestion mechanism of native starch has been reported to be quite complex as it is associated with several interconnected factors, including the granule architecture (such as granule size, surface pores, etc.), crystalline structure (crystalline type and relative crystallinity), and molecular structure (such as amylose content, chain length distribution, etc.) [20]. The significantly lower digestion rate of Caopu (6.6 × 10^−3^ min^−1^) might be attributed to its higher amylose content (30.1%, given in Table 2) compared with other starch. It is generally agreed that the amylose content is positively correlated with the resistance of native starch to enzyme hydrolysis because amylose could enhance the integrity of starch granules by intertwining with amylopectin crystallites. This could, therefore, hinder the penetration of digestion enzymes into starch granules, resulting in a lower digestion rate [21,22,23]. Normal maize starch, however, had a significantly higher digestion rate (8.5 × 10^−3^ min^−1^) than Caopu, although its amylose content (29.1%) was similar to Caopu. This is probably because of the porous structure of maize starch granules, while longan seed starch showed a smooth surface as shown in the SEM images displayed in Figure 1. The pores on the granule surface of maize starch link to the hilum of the starch granule through interior channels, which could improve the accessibility of digestion enzymes to the interior of the starch granules [24]. In addition, the digestion rate of Shixia longan seed starch (8.9 × 10^−3^ min^−1^) was similar to normal maize starch, whereas that of Chuliang (7.7 × 10^−3^ min^−1^) was significantly lower, although the amylose content of the Chuliang seed starch was similar to Shixia. This could be explained by the higher crystallinity of Chuliang (29.2%) compared with Shixia (28.6%), resulting in a more intact structure which is more difficult for enzymes to digest.

In terms of freshly gelatinized starch samples, much quicker digestion processes were detected, as shown in Figure 4B. All samples were rapidly digested in the first 30 min of digestion, and the amount of starch digested became relatively stable after 1 h of digestion. Similar digestion curves were observed for all the samples, indicating that the digestion process of the freshly gelatinized longan seed starch of the three cultivars and the maize starch are similar. Not surprisingly, the digestion rate coefficient values were not significantly different among the samples. This is in agreement with results reported by Wang et al. [14] who found no significant difference among starch from different plant varieties within the same species nor different species. This suggests that for fully cooked starch in foods, the starch source or structure does not have a significant impact on the digestion properties of starch.

## 3. Materials and Methods

### 3.1. Materials

Normal maize starch was purchased from Tiancheng Corn Development Co. Ltd., Siping, China. Chuliang and Shixia longan seeds were collected from local orchards in Maoming Gaozhou (Guangdong, China) in July 2017, while Caopu longan seeds were from orchards in Chaozhou (Guangdong, China) in July 2017.

Pancreatin from porcine pancreas was purchased from Sigma-Aldrich Pty. Ltd. (St. Louis, MO, USA). Amyloglucosidase (from *Aspergillus niger*), isoamylase (from *Pseudomonas sp.*), total starch (AA/AMG) assay kit, and D-glucose (GOPOD Format) kit were purchased from Megazyme International, Ltd. (Bray Co., Wicklow, Ireland). Pullulan standards with known peak molecular weights were purchased from Polymer Standards Service (PSS) GmbH (Mainz, Germany). Dimethyl sulfoxide (DMSO, GR grade for analysis) was purchased from Merck Co. Inc. (Kenilworth, NJ, USA). Other chemicals were reagent-grade and used as received.

### 3.2. Composition of Longan Seeds

The moisture content was determined by drying the samples in an oven at 110 °C overnight and recording the weight loss of moisture. Longan seeds were ground into a powder using a grinder, and then the starch content was analyzed using a total starch (AA/AMG) assay kit. The crude protein content was estimated from the nitrogen content of longan seeds using a Kjeltec instrument (Kjeltec-8200, Foss Electric, Hilleroed, Denmark) with a conversion factor of 5.18 [25]. The crude lipid content was analyzed by Soxhlet extraction following AOAC method 920.39C [26].

### 3.3. Isolation of Starch from Longan Seeds

Starch was isolated from longan seeds following the wet-milling method described by Wang et al. [27]. Longan seeds (40 g) were first steeped in 120 mL of sodium bisulfite solution (0.45% sodium metabisulfite, *w*/*v*) overnight, after which they were ground into a slurry using a homogenizer (T18 basic ULTRA-TURRAX, IKA, Wilmington, NC, USA). The milled longan seed slurry was filtered through a 106 µm screen. The residues remaining on the screen were collected and mixed again with sodium bisulfite solution. The mixture was homogenized and filtered again. These steps were repeated until the residue quality no longer decreased. All filtrate fractions were combined and then stirred for 1.5 h with a mixed solution containing NaCl solution (0.1 M) and toluene at a ratio of 9:1. The mixture was left to stand until the starch granules had settled at the bottom. The upper toluene layer and NaCl solution were removed. The NaCl solution/toluene steps were repeated until the toluene layer became clear. The isolated starch samples were washed with water and then with ethanol three times, followed by a drying step in a 40 °C oven.

### 3.4. Starch Morphology

Starch samples were spread onto a circular metal stub covered with double-sided adhesive tape and were coated with platinum by a sputter coater (Cressington, UK). Images of starch granules were acquired using a scanning electron microscope (SEM, model EVO 18, Zeiss, Germany) under an accelerating voltage of 10 kV and at multiple magnifications [28].

### 3.5. X-ray Diffraction

X-ray patterns were performed with an X-ray diffractometer (D8 Advance, Bruker, Germany) at 44 kV and 27 mA Cu-Ka radiation. Diffractograms were obtained from 3–45° (2θ) at a scan rate of 5°/min. Starch samples were equilibrated in a saturated relative humidity chamber for 24 h at 25 °C before analysis [29]. The relative crystallinity was calculated as the ratio of the crystalline peak area to the total diffraction using PeakFit software (Version 4.0, Systat Software Inc., San Jose, CA, USA).

### 3.6. Molecular Structure

The CLDs of isolated longan seed starch were characterized using an Agilent 1260 Infinity size exclusion chromatography (SEC) system (Agilent, Santa Clara, CA, USA) with a refractive index detector (RID, Optilab UT-rEX, Wyatt, Santa Barbara, CA, USA) following a previously published method [30]. Starch was firstly debranched using isoamylase. The debranched starch samples were then freeze-dried and dissolved in DMSO containing 0.5% (*v*/*v*) solution (DMSO/LiBr solution) before being injected into the SEC system with DMSO/LiBr solution at a flow rate of 0.6 mL/min. Size separation of the starch chains was performed using a pre-, Gram 100, and Gram 1000 columns (Polymer Standards Service, Mainz, Germany) at 80 °C. Amylose content of the longan seed starch was calculated from the CLDs of each starch as the ratio of the area under the curve of amylose CLD to that of the whole CLD curve.

### 3.7. Thermal Properties

Starch (~4 mg, dry basis) was weighed in a high-pressure pan and then distilled water (three times the weight of the starch) was added. The pan was immediately sealed and allowed to equilibrate at room temperature for 1 h. Each crucible was loaded in a DSC (DSC-8000, PerkinElmer, Norwalk, CT, USA) and then scanned from 30 to 110 °C at a heating rate of 10 °C/min [31]. The enthalpy change (ΔH), onset (T_o_), peak (T_p_) and conclusion (T_c_) temperatures were calculated using Pyris software (PerkinElmer, Norwalk, CT, USA).

### 3.8. In Vitro Starch Digestibility

In vitro starch digestion of raw and cooked longan seed starch was carried out using pancreatin and amyloglucosidase following a previously described method with modifications [14]. Raw longan seed starch (100 mg, dry basis) was dispersed in 2 mL of distilled water and then digested with 2 mg of pancreatin and 100 μL of amyloglucosidase in 7.9 mL of sodium acetate buffer solution (0.2 M, pH 6.0, containing 200 mM CaCl_2_, 0.49 mM MgCl_2_, and 0.02% NaN_3_) at 37 °C. Digesta (0.1 mL) was collected at 0, 30, 60, 90, 120, 180, 240, 300, 360, 420, 480, and 540 min, after which 0.9 mL of absolute ethanol was immediately added to deactivate the enzymes. In terms of cooked starch digestion, the isolated longan seed starch (50 mg, dry basis) was first gelatinized in 2 mL of distilled water by cooking in a boiling water bath for 30 min. After cooling down, each freshly gelatinized starch was digested using 0.25 mg of pancreatin and 12.5 μL of amyloglucosidase in a sodium acetate buffer solution following a procedure similar to that for raw starch, except that starch digesta (0.1 mL) was collected at 0, 20, 30, 60, 120, 180, 240, and 300 min.

The digestibility of both raw and cooked starch samples was calculated from the content of glucose released during digestion. The digestogram was then fitted to first-order kinetics using logarithm of the slope (LOS) plot following the equation:

ln(dC_t_/dt) = −kt + ln(C_∞_k) (1)

where C_t_ is the percentage of starch digested at time t (min), C_∞_ is the estimated percentage of starch digested by the end of digestion, and k is the digestion rate coefficient [32,33].

### 3.9. Statistical Analysis

Results were expressed as the mean and standard deviation of at least duplicate measurements. Analysis of variance (ANOVA) with the general linear model and Tukey’s pairwise comparisons were used to analyze data in Minitab 16 (Minitab Inc., State College, PA, USA). Significant differences of the mean values were determined at *p* < 0.05.

## 4. Conclusions

Longan seeds of Chuliang, Shixia, and Caopu cultivars are rich in starch with contents ranging from 44.9–49.5%. The seed starch of the three longan cultivars had similar granule shape and size, with most granules having smooth surfaces and an oval or irregular polygonal shape. The CLDs characterized using SEC showed differences among the seed starch of the three longan cultivars, although the same crystalline type (A-type) was detected with the relative crystallinity varying from 28.9 to 29.2%. Chuliang longan seed starch had the highest proportion of amylopectin chains with DP 30–100, leading to its relatively higher crystallinity than the others. The starch also differed in its amylose structure. The amylose content of longan seed starch varied with longan cultivar, with Caopu exhibiting a significantly higher value (30.1%) than the Shixia (26.6%) and Chuliang (26.7%) cultivars. This contributes to the lower gelatinization temperatures and enthalpy change of Caopu than the others. For raw longan seed starch, the digestion rate differed significantly with cultivar, which is attributed to their distinct molecular and crystalline structures. After full gelatinization, however, the digestion profile of the seed starch of the longan cultivars became similar, suggesting that the digestion process remains the same no matter the starch structure, botanical source, or growth.

## Figures and Tables

**Table 1:** Composition of longan seeds from various cultivars ^1^.

Cultivar	Starch Content (%)	Protein Content (%)	Fat Content (%)
Chuliang	44.9 ± 0.4 ^a^	6.0 ± 0.0 ^c^	3.4 ± 0.2 ^a^
Shixia	46.2 ± 1.5 ^a^	8.1 ± 0.0 ^a^	2.0 ± 0.0 ^b^
Caopu	49.5 ± 1.6 ^a^	7.6 ± 0.0 ^b^	2.5 ± 0.0 ^b^

^1^ Mean ± standard deviation was obtained from duplicate measurements. Values with different letters in the same column show significant difference at *p* < 0.05.

**Table 2:** Amylose content and thermal properties of longan seed starch from various cultivars in comparison with normal maize starch ^1^.

Source	Amylose Content (%)	T_o_	T_p_	T_c_	∆H
		(°C)	(°C)	(°C)	(J/g)
Chuliang	26.7 ± 0.9 ^b^	72.3 ± 0.4 ^b^	80.5 ± 0.1 ^a^	88.3 ± 0.3 ^a^	16.4 ± 0.4 ^a^
Shixia	26.6 ± 0.3 ^b^	72.8 ± 0.1 ^b^	79.0 ± 0.1 ^a,b^	85.6 ± 0.0 ^b^	15.6 ± 0.2 ^a^
Caopu	30.1 ± 0.8 ^a^	69.6 ± 0.4 ^c^	76.7 ± 0.3 ^b^	84.4 ± 0.5 ^c^	13.5 ± 0.5 ^b^
Maize	29.1 ± 0.2 ^a,b^	75.4 ± 0.8 ^a^	79.2 ± 0.9 ^a,b^	88.0 ± 0.0 ^a^	10.3 ± 0.6 ^c^

^1^ Mean ± standard deviation was obtained from duplicate measurements. Values with different letters in the same column show significant difference at *p* < 0.05.

**Table 3:** The digestion rate coefficient (k) of raw and freshly gelatinized longan seed starch and normal maize starch ^1^.

Source	k (10^−3^ min^−1^)	
	Raw Starch	Gelatinized Starch
Chuliang	7.7 ± 0.2 ^b^	45.2 ± 2.8 ^a^
Shixia	8.9 ± 0.4 ^a^	45.1 ± 1.1 ^a^
Caopu	6.6 ± 0.0 ^c^	44.3 ± 0.6 ^a^
Maize	8.5 ± 0.0 ^a,b^	45.8 ± 0.6 ^a^

^1^ Mean ± standard deviation was obtained from duplicate measurements. Values with different letters in the same column show significant difference at *p* < 0.05.
